# Ten-fold Robust Expansion Microscopy

**DOI:** 10.21769/BioProtoc.4698

**Published:** 2023-06-20

**Authors:** Hugo G. J. Damstra, Boaz Mohar, Mark Eddison, Anna Akhmanova, Lukas C. Kapitein, Paul W. Tillberg

**Affiliations:** 1 Cell Biology, Neurobiology and Biophysics, Department of Biology, Faculty of Science, Utrecht University, Utrecht, The Netherlands; 2 Janelia Research Campus, HHMI, Ashburn, Virginia 20147, USA

**Keywords:** Expansion microscopy, Super resolution, Light microscopy, Antibody labeling, Ultrastructure, Protein stain

## Abstract

Expansion microscopy (ExM) is a powerful technique to overcome the diffraction limit of light microscopy that can be applied in both tissues and cells. In ExM, samples are embedded in a swellable polymer gel to physically expand the sample and isotropically increase resolution in x, y, and z. By systematic exploration of the ExM recipe space, we developed a novel ExM method termed Ten-fold Robust Expansion Microscopy (TREx) that, as the original ExM method, requires no specialized equipment or procedures. TREx enables ten-fold expansion of both thick mouse brain tissue sections and cultured human cells, can be handled easily, and enables high-resolution subcellular imaging with a single expansion step. Furthermore, TREx can provide ultrastructural context to subcellular protein localization by combining antibody-stained samples with off-the-shelf small molecule stains for both total protein and membranes.

## Background

Expansion microscopy (ExM) circumvents the diffraction limit of light microscopy by physically expanding the specimen four-fold in each dimension (Chen et al., 2015; Tillberg et al., 2016). Expansion is achieved by chemically anchoring proteins and other biomolecules directly to a hyper-swelling gel, followed by aggressive proteolysis to enable uniform swelling of the gel material. Assuming sufficiently high labeling density, the resolution increase of ExM depends on the expansion factor of the gel recipe used. Recently, ExM variants have been described that seek to improve resolution by increasing the expansion factor, for example by multiple rounds of expansion such as iterative ExM (Chang et al., 2017), by decreasing the concentration of crosslinker (Chen et al., 2015), usually bisacrylamide (bis), or by using a different crosslink chemistry (Truckenbrodt et al., 2019). By systematic exploration of the expansion gel recipe space, we assessed the limits of single-round expansion and generated Ten-fold Robust Expansion Microscopy (TREx), an optimized ExM method that allows for robust ten-fold expansion in a single step. Since TREx uses the same chemistry and single-round embedding procedure as the original ExM protocol, it can be easily implemented for users already familiar with ExM. Moreover, we show ten-fold expansion sufficiently de-crowds biological specimens to visualize local protein densities using general protein and membrane stains.

## Materials and reagents

1. 4-Hydroxy-TEMPO (4HT) (Sigma-Aldrich, catalog number: 176141)
2. Acrylamide 40% solution (Sigma-Aldrich, catalog number: 01697)
3. Acrylic acid (Sigma, catalog number: 147230)
4. Acryloyl-X SE (AcX) (Thermo Fisher, catalog number: A20770)
5. Ammonium persulfate (APS) (Sigma-Aldrich, catalog number: 215589)
6. Bovine serum albumin (BSA) (Sigma-Aldrich, catalog number: A9647)
7. N,N'-Methylenebisacrylamide (bis) 2% solution (Fisher Scientific, catalog number: BP1404)
8. Coverslips (18 mm) (Marienfeld, catalog number: 107032)
9. DAPI (Thermo Fisher, catalog number: D1306)
10. DMSO (Thermo Fisher, catalog number: D12345)
11. Gluteraldehyde (GA) (Electron Microscopy Sciences, catalog number: 16100)
12. Guanidine HCl (Sigma, catalog number: G3272)
13. Methanol (MeOH) (Sigma-Aldrich, catalog number: 34860)
14. Multi-well culture plates (6-well and 12-well) (Sigma-Aldrich, catalog numbers: CLS3335 and CLS3336, respectively)
15. Paraformaldehyde (PFA) (Electron Microscopy Sciences, catalog number: 15710)
16. Proteinase K (Thermo Fisher, catalog number: EO0491)
17. Silicone sheet material (Sigma-Aldrich, catalog number: GBL664107/GBL665501)
18. Sodium acrylate (Sigma-Aldrich, catalog number: 408220)
19. Sodium dodecyl sulfate (SDS) (Sigma, catalog number: 71736)
20. Tetramethyl ethylenediamine (TEMED) (Bio-Rad, catalog number: 1610800)
21. Triton X-100 (Sigma, catalog number: T8787)
22. PBS 10× (Thermo Fisher, catalog number: 70011044)
23. TAE 50× (Thermo Fisher, catalog number: B49)
24. NaCl (Thermo Fisher, catalog number: 447302500)
25. Tris (Sigma, catalog number: T1503)
26. Gelation solution (see Recipes)
27. Digestion buffer (see Recipes)
28. Gelation solution recipe for tissue slices (see Recipes)
29. Disruption buffer (see Recipes)

## Procedure

**Part I: Protocol for cultured cells**

- **Fixation**
  1. Fix cells (18 mm coverslips, coated for optimal cell growth as desired) for 10 min at 37 °C using pre-warmed fixative. Fixative may be 4% PFA, 4% PFA + 0.1% glutaraldehyde (GA) for preservation of membranes, -20 °C MeOH, or extraction as described in Damstra et al. (2022).
  2. Wash with PBS twice.
  3. If no permeabilization will be performed (e.g., if no immunofluorescence IF labeling will be performed), permeabilize for 10 min with 0.1% Triton X-100 in PBS at room temperature (RT) (this is important to ensure gel spans membranes fully), followed by washing with PBS three times.
- **IF (optional, most standard IF methods may be used)**
  1. Block with 3% BSA in PBS for 1 h at RT.
  2. Incubate with primary antibodies in 3% BSA in PBS for 1–3 h at RT or overnight at 4 °C.
  3. Wash with PBS three times.
  4. Incubate with secondary antibodies in 3% BSA in PBS for 1–3 h at RT or overnight at 4 °C.
  5. Wash with PBS three times.
- **Anchoring**
  1. Incubate with 0.1 mg/mL AcX in PBS overnight at RT or 0.2 mg/mL for 1 h at RT.
    For AcX stock solution, dilute 5 mg of AcX in 500 μL of anhydrous DMSO (10 mg/mL), aliquot, and store at -20 °C (dilute a 20 μL aliquot in 2 mL of PBS just prior to use).
- **Gelation**
  1. Assemble gelation chamber [parafilm-covered glass slide and silicone ring (13 mm diameter)] and pre-chill on ice (Figure 1).
    **Figure 1.**
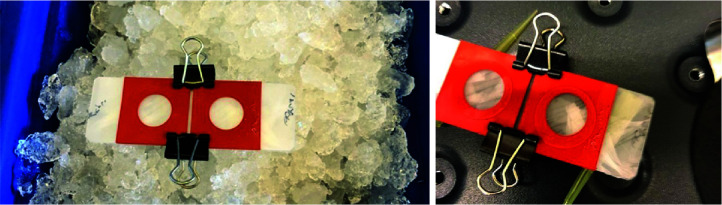
Gelation chamber before (left) and during gelation (right). Gelation chamber consists of a parafilm-covered glass slide and a silicon spacer that is held in place by binder clips. The gelation chamber is formed when the coverslip that contains the cells is placed cell-side down on the silicon spacer (right) ensuring contact with the gelation solution.
  2. Prepare monomer solution [gelation solution (see Recipes) without the APS and TEMED] and keep on ice. Monomer solution can optionally be aliquoted and stored at -20 °C. When using an aliquot, please ensure the aliquot is fully thawed at RT because acrylamide has reduced solubility at low temperatures.
  3. Wash cells with PBS once.
  4. Add TEMED and APS to the monomer solution to form gelation solution, vortex thoroughly, and put back on ice.
  5. Transfer 170 μL to the chamber (pipette directly on the parafilm), tap the side of coverslip on a tissue to remove excess PBS, and put cells down on the monomer solution to close the chamber. Transfer directly to the incubator set at 37 °C for 1 h (Figure 1).
- **Homogenization**
  1. Gently remove gels from the gelation chamber and transfer to a 12-well plate. At this point, the gel typically remains attached to the coverslip. The gel will spontaneously separate from the coverslip during either enzymatic or non-proteolytic homogenization. For enzymatic digestion: add 2 mL of digestion buffer (see Recipes) and digest for 4 h at 37 °C. Optional: add DAPI to digestion buffer.
    Depending on the experiment, a non-proteolytic disruption (3 h incubation in 5% SDS, 200 mM NaCl, and 50 mM Tris pH 7.5 at 80 °C) or a hybrid disruption and digestion approach can be used to increase the amount of protein retained after digestion (see below). For staining with general protein stains in combination with digestion, we typically wash the gel twice for 15 min with PBS, incubate with 20 μg/mL of fluorescently labeled NHS-ester or maleimide for 1.5 h at RT, and then proceed with digestion. When using disruption or a hybrid approach to increase the number of accessible residues for labeling, general protein stains can be added after the disruption step.
- **Expansion**
  1. Transfer gel to a 15 cm dish and completely fill with water (Milli-Q). Exchange water at least twice after 30 min and leave overnight at RT to expand (Figure 2a).
    **Figure 2.**
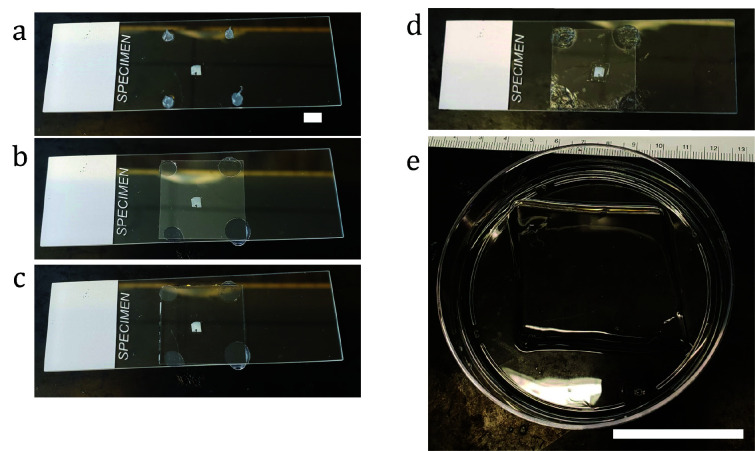
Illustration of gel chamber construction for expansion of tissue and example of gel expansion. a) Piece of tissue positioned on glass slide between dabs of vacuum grease. b) Coverslip pressed down gently to contact the tissue, held in place by the dabs of vacuum grease. c) Completed chamber, filled with gelation solution. d) After gelling at 37 °C, the coverslip is gently removed and flipped over, and excess gel is trimmed away from the specimen using a curved scalpel. e) After digestion and washing in water, the fully expanded gel. Scale bars: (a) 0.5 cm, (b) 5 cm.

**Part II: Protocol for expanding tissue**

- **Perfusion and post-fixation**
  1. Mice were transcardially perfused with ice-cold 4% formaldehyde in 100 mM sodium phosphate buffer, pH 7.4. Brains were dissected out and post-fixed in 4% formaldehyde between 2 h and overnight at 4 °C, followed by washing with PBS and slicing by vibratome at 100–300 μm.
  2. Wash with PBS twice.
  3. If no permeabilization will be performed (e.g., for IF), permeabilize for 30 min with 0.1% Triton X-100 in PBS at RT (this is important to ensure gel spans membranes fully), followed by washing with PBS three times.
- **IF (optional, most standard IF methods may be used)**
  1. Block with blocking buffer (2% BSA, 0.1% Triton X-100 in PBS) at RT for 30 min to 2 h.
  2. Incubate with primary antibodies in blocking buffer at RT for several hours to several days.
  3. Wash with blocking buffer four times for 30 min.
  4. Incubate with secondary antibodies in blocking buffer at RT for several hours to several days.
  5. Wash with blocking buffer three times for 30 min and with PBS once.
- **Anchoring**
  1. Treat tissue slices with 0.1 mg/mL AcX in PBS overnight or 0.2 mg/mL for 1 h.
    For AcX stock solution, dilute 5 mg of AcX in 500 μL of anhydrous DMSO (10 mg/mL), and store at -20 °C (dilute 20 μL aliquot in 2 mL of PBS just prior to use).
  2. Wash with PBS.
- **Gelation**
  1. Unlike for cultured cells, tissue slices must be incubated with gelation solution (including APS, TEMED, and the radical inhibitor 4HT to delay the onset of gelation; see Recipes) to ensure the monomer solution can completely diffuse through the tissue. Prepare monomer solution (gelation solution without APS, TEMED, or 4HT), which can optionally be aliquoted and stored at -20 °C. When using an aliquot, please ensure the aliquot is fully thawed at RT because acrylamide has reduced solubility at low temperatures.
  2. For the gelation chamber, place four dabs of vacuum grease on a glass slide, spaced to coincide with the corners of a glass coverslip.
  3. On ice, add gelation solution to the specimen and incubate on ice for 20 min while shaking.
  4. Remove excess gelation solution and transfer the specimen to glass slide in the middle of the dabs of vacuum grease. To seal the chamber, place a glass coverslip on top of the dabs of vacuum grease and gently press down until the coverslip contacts the tissue.
  5. Gently pipette gelation solution in excess from the side, making sure the specimen remains at least ~3 mm away from air or silicone grease, and transfer directly to incubator set at 37 °C for 1 h.
- **Homogenization**
  1. Gently remove gel from gelation chamber by teasing the coverslip off with a razor blade and tweezers.
  2. Trim the gel close to the specimen and wash once in PBS for 5 min.
  3. Disrupt with proteinase K as with cultured cells.
  4. Wash with PBS four times for 30 min each.
    To preserve the most total protein for NHS-ester staining, the anchoring and disruption steps may be reduced in strength, though potentially at the cost of reduced expansion isotropy between different subcellular structures. For example, both the AcX and proteinase K concentrations can be reduced ~10-fold, with the digestion step followed by high-temperature non-proteolytic disruption with SDS, by transferring the gel to an excess volume of disruption buffer and incubating for 3 h at 80 °C.
    If cracks appear in the specimen upon expansion, increasing the proteinase K concentration or time in disruption buffer may help. If cracks persist, increasing the temperature of disruption buffer treatment (e.g., to 90 °C) can greatly increase the strength of disruption. The original ExM disruption method using proteinase K diluted 1:100 in buffer containing guanidium can also be tried if more aggressive digestion is needed. To add sub-cellular context, disrupted specimens may be stained with NHS-ester dyes, e.g., Atto594 or Alexa488, diluted to 20 μg/mL in 1× PBS for 1 h at RT.
- **Expansion**
  1. Transfer gel to a 6-well plate or 15 cm dish, wash with water for 30 min at least three times, and leave overnight at RT to expand. Keep covered to protect from acidification due to CO_2_ in room air.

## Notes

**Fine tuning expansion factor**

Increasing the bisacrylamide concentration will decrease the expansion factor and make the gel more rigid; decreasing the bisacrylamide concentration will increase the expansion factor at the expense of mechanical integrity. If ten-fold expansion is not obtained, we recommend varying the bisacrylamide concentration accordingly. If ten-fold expansion is not required, bisacrylamide can be increased to improve the toughness of the gel at the expense of expansion factor. Of note, we also observed the freshness of APS can have a moderate impact on the expansion factor. We recommend aliquoting APS and not subjecting stocks to multiple rounds of freezing to prevent this source of variation.

**Sodium acrylate purity**

We have observed commercial sodium acrylate comes in varying purity with unknown impact on gel quality. 4M sodium acrylate can also be made by neutralizing acrylic acid with 10 N NaOH. The protocol for 20 mL of sodium acrylate (4 M) is as follows:

1. In a fume hood, combine 5.5 mL of acrylic acid and 4.5 mL of water (Milli-Q). Next, gradually add 7.2 mL of NaOH (10 M). Use a water bath to prevent excessive heating. After most of the highly volatile and noxious acrylic acid has been converted to non-volatile sodium acrylate, the solution can be transferred out of the fume hood.
2. Add 1 M NaOH gradually until the pH is 7.5–8 (usually around 1 mL). Use a pH meter to monitor pH, not pH test strips. Buffering capacity of 4 M acrylate at pH 7.75 is only ~4 mM. Add water up to a final volume of 20 mL.

**Imaging methods**

TREx is compatible with any microscope. Confocal and light-sheet microscopy can be used to take particular advantage of the compatibility of TREx with thick tissue specimens. Since the expanded hydrogel is mostly water, it is particularly compatible with water objectives. A good compromise of long working distance and high numerical aperture (NA) is important because expansion renders specimens both thicker and spatially diluted. We typically use 40–86× water immersion with NA 1.0–1.2, or for thicker specimen air or water immersion objectives with NA 0.8–1.0. To prevent drift during acquisition, expanded gels may be immobilized during imaging by coating the glass support, such as a well plate or imaging chamber, with poly-L-lysine, which will adhere strongly to the expanded gel. For quick acquisitions, immobilization is not strictly necessary.

**General protein stains**

TREx is compatible with any maleimide or NHS-ester general protein stain. The labeling pattern is modulated by the hydrophobicity of the fluorophore (Sim et al., 2021); thus, we recommend the user to test a range of dyes. We typically use Alexa 488/594 NHS (Thermo Fisher, A20100, A37572, respectively), Cyanine3/Cyanine5 NHS (Lumiprobe, 11020, 13020, respectively), or Atto 647N maleimide (Sigma-Aldrich, 05316).

**Gelation chambers**

**Figure 3.**
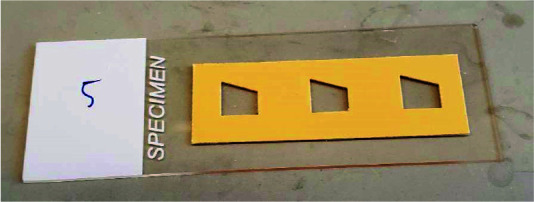
Gelation chamber for cultured cells assembled using adhesive silicone sheet material from Digi-key, with right trapezoid chambers cut by laser cutter

This protocol is compatible with many gelation chamber designs used for different types of specimens. As a generic starting point for cultured cells, we recommend using an uncharged glass slide as the chamber bottom and adhesive silicone material as a spacer gasket. Positive charged glass can also be used—this surface will stick to the gel more strongly. For spacer gaskets, silicone isolators (e.g., Sigma GBL664107 or GBL665501) are an easy solution (Figure 3). The silicone sheet material from Digi-key comes as thin as 250 μm, allowing for faster gel expansion, and can be trimmed to make a gasket of any shape (e.g., a right trapezoid, for keeping track of gel orientation) using a laser cutter or other cutting method. The chamber top piece can be a coverslip (possibly with adherent cells) or a glass slide. For tissue slices up to at least 300 μm thick, we simply sandwich the tissue between a glass slide and a glass coverslip, with dabs of vacuum grease at the corners of the coverslip to keep it in place. Keep in mind that the portion of the gel that forms within ~3 mm of any silicone or air edge should be trimmed away and discarded, as oxygen from these materials inhibits polymerization, altering the process of gelation.

## Recipes

1. **Gelation solution**
  **Table:** 

Reagent	Stock	Final concentration	Amount
Sodium acrylate	4 M	1.1 M	271 μL
Acrylamide	5.6 M	2 M	360 μL
Bis	2%	0.005%	2.5 μL
PBS	10×	1×	100 μL
Water (Milli-Q)			236.5 μL
TEMED	10%	0.15%	15 μL
APS	10%	0.15%	15 μL
  Sodium acrylate 4 M: dissolve 0.38 g/mL in Milli-Q or by neutralizing acrylic acid (see below).
  Acrylamide 5.6 M equals 40% stock solution
2. **Digestion buffer**
  **Table:** 

Reagent	Stock	Amount per gel
TAE buffer		1,550 μL
Triton X-100	10% in PBS	100 μL
Guanidine HCl	5 M	320 μL
Proteinase K	600 U/mL	30 μL
3. **Gelation solution recipe for tissue slices**
  **Table:** 

Reagent	Stock	Final concentration	Amount
Sodium acrylate	4 M	1.1 M	2.7 mL
Acrylamide	5.6 M	2.0 M	3.6 mL
Bis	2%	0.005%	0.025 mL
PBS	10×	1×	1.0 mL
Water (Milli-Q)			2.225 mL
TEMED	10%	0.15%	0.15 mL
APS	10%	0.15%	0.15 mL
4HT	0.1%	0.0015%	0.15 mL
  Acrylamide 5.6 M equals 40% stock solution
4. **Disruption buffer**
  **Table:** 

Reagent	Stock	Final concentration	Amount
SDS	10%	5%	10 mL
Tris pH 7.5	1 M	0.05 M	1 mL
NaCl	5 M	0.2 M	0.8 mL
Water (Milli-Q)			8.2 mL
